# Xpert MTB/RIF assay can be used on archived gastric aspirate and induced sputum samples for sensitive diagnosis of paediatric tuberculosis

**DOI:** 10.1186/s12866-015-0528-z

**Published:** 2015-09-29

**Authors:** Sarman Singh, Amit Singh, Suneel Prajapati, Sushil K. Kabra, Rakesh Lodha, Aparna Mukherjee, Varinder Singh, Anneke C. Hesseling, Harleen M. S. Grewal, Delhi Pediatric TB study group

**Affiliations:** Division of Clinical Microbiology and Molecular Medicine, Department of Laboratory Medicine, All India Institute of Medical Sciences, Ansari Nagar, New Delhi 110 029 India; Department of Paediatrics, All India Institute of Medical Sciences, Ansari Nagar, New Delhi 110 029 India; Department of Paediatrics, Kalawati Saran Children Hospital, New Delhi, 110 001 India; Paediatric Research, Desmond Tutu TB Centre, University of Stellenbosch, Stellenbosch, South Africa; Department Clinical Science, Infection, University of Bergen, Bergen, Norway; Department of Microbiology, Haukeland University Hospital, Bergen, N-5021 Norway

**Keywords:** Pediatrics Tuberculosis, India, Gastric Aspirate, Induced Sputum, Archived Samples, mPCR, MGIT Culture, Xpert MTB/RIF

## Abstract

**Background:**

Tuberculosis (TB) in children is neglected, mainly due to lack of sensitive diagnostic tools. Recently Xpert MTB/RIF assay has revolutionized the diagnostic field, but its usefulness in pediatric TB has not been reported from India and no report is available on its use on long term archived samples.

**Methods:**

We recruited 130 pediatric patients with probable intrathoracic tuberculosis and their gastric aspirate (GA) and induced sputum (IS) samples on 2 consecutive days were collected between January 2009 and December 2012. All samples (n = 520) were subjected to smear examination, BACTEC-MGIT culture and *in-house* multiplex PCR. An aliquot of each sample was stored at −80 °C and tested in Xpert MTB/RIF assay in 2013.

**Results:**

Sample wise and patient wise detection rate of smear microscopy was 4.4 % and 10 %, while for BACTEC-MGIT culture this rate was 24.4 % and 46.9 %, respectively. Of the 130 day 1 GA samples, 31.5 % and 27.7 % day 2 GA samples were culture positive. Only 17.7 % GA samples were positive on both days. Of the 130 IS samples collected on day 1 and day 2, 15.4 % and 23.1 % samples were culture positive. A combination of GA and IS yielded best results. Combining both GA and IS, the overall sensitivity of Xpert MTB/RIF on smear and culture positive samples was 95.6 %. In smear negative and culture positive samples its sensitivity was 62.5 %. The duration of sample storage impacted the Xpert MTB/RIF test performance (p = 0.0001). In smear positive samples stored for 650–849 days, its sensitivity was 85.7 % and 77.1 % for IS and GA samples which dropped to 33.3 % and 50 %, respectively, if stored for more than 1050 days.

**Discussion:**

Confirmatory diagnosis of tuberculosis particularly in children is a medical challenge. No laboratory or radiological test can reach to a satisfactory level of diagnostic sensitivity. However, in this study we foundthat combination of multiple samples and multiple diagnostic tests can give much better yield, though notoptimum. In present study, combination of 2 gastric aspirates (GA) and 2 induced sputum (IS) samples collected on two consecutive days, and tested on three diagnostic methods yielded a significantly high detection rate. Despite long term storage, the overall sensitivity of Xpert MTB/RIF on smear and -culture positive samples remained very high. But after storing these samples under subfreezing conditions thesensitivity of Xpert MTB/RIF decreased significantly. This is expected because even if the sample is smear and culture positive, the count of surviving mycobacteria goes down, after several years this count can reach to a undetectable level.

**Conclusion:**

This report shows that smear and culture positive samples stored at subfreezing conditions for several years can be used in the Xpert MTB/RIF assay, while maintaining appreciable diagnostic test sensitivity and specificity.

## Background

World Health Organization (WHO) estimated the global burden of tuberculosis (TB) at 9 million new cases and 1.5 million deaths in 2013 [1]. This included up to 15 % burden of pediatric cases. About 74,000 children die of TB every year and there are around half a million new cases annually [2]. Like in adults, the majority (70–80 %) of child TB cases present with pulmonary tuberculosis (PTB). Tuberculosis in children has been relatively neglected, mainly due to challenges in the availability of effective diagnostic tools [3]. India is 17th among 22 high burden countries in terms of overall TB incidence rate, but very little information is available regarding epidemiology of TB in children, its diagnosis and management [4, 5].

In most settings diagnosis of pediatric TB is made on the basis of contact tracing, and very few attempts have been made for active case detection. This is mainly due to lack of a pathognomonic clinical presentation in pediatric TB and lack of sensitive diagnostic tools [3, 4, 6, 7].

With limited tools for confirmatory laboratory diagnosis, evaluation of medical history, tuberculin skin testing, chest x-ray and a lack of response to antibiotics help in making the clinical diagnosis. Smear microscopy and microbiological confirmation is rarely achieved due to poor sensitivity of these tests, in part due to the paucibacillary nature of the disease and inability of young children to provide an optimal sputum sample [6]. Alternate samples include gastric aspirate (GA), induced sputum (IS), nasopharyngeal aspirate, bronchio-alveolar lavage, laryngeal swab, string test and stool samples [3, 7–10]. Depending on the diagnostic test used, these samples have shown variable sensitivity and specificity. It has been reported that gastric lavage samples provide the highest (40–92 %) detection rate, depending on the sensitivity of the laboratory test adopted. Other samples have shown poorer detection rates ranging from 4–43 % for bronchoalveolar lavage, 24–30 % for nasopharyngeal aspiration, 27–63 for laryngeal swab and 20–30 % by using induced sputum. Some workers have also used string test but results have not been encouraging [3, 7, 11–13].

The recent introduction of the Xpert MTB/RIF assay (Cepheid, USA) has significantly transformed the diagnostics of tuberculosis in adults, but its application for the diagnosis of pediatric TB is under evaluation. This assay is rapid and provides results within 2 h [14]. In a policy statement World Health Organization (WHO) in 2011, recommended the use of Xpert MTB/RIF assay as a preliminary diagnostic tool among children, in adults with suspected human immunodeficiency virus (HIV) associated tuberculosis and in all MDR TB suspected cases [15]. Subsequently, several workers have started using Xpert MTB/RIF assay for the diagnosis of pediatric tuberculosis [10, 16–18]. However, the wide spread use of Xpert MTB/RIF for the diagnosis of pediatric TB on a routine basis still remains a distant option. To date, there are only a few studies on the application of the Xpert MTB/RIF for the diagnosis of pediatric TB. Two studies report on the use of the Xpert MTB/RIF on induced sputum two on its use in gastric lavage samples. There are no studies that report on use of Xpert MTB/RIF in both GA and IS samples simultaneously on the same patient. Further, there is no study from India on the evaluation of Xpert MTB/RIF for the diagnosis of pediatric pulmonary TB. Moreover, the utility of Xpert MTB/RIF on archived GA and IS samples has previously not been examined. Since the Xpert MTB/RIF became available only after 2011, there is no other rapid test which can detect TB and RIF resistance simultaneously in samples which were collected before 2011. This study also examines the effect of long term storage of clinical samples on for the detection of TB by the Xpert MTB/RIF assay.

We have earlier reported that GA samples provide better yields than IS samples [19]. In a subsequent report we also showed that neutralizing the pH of gastric aspirate before processing leads to a higher rate of contamination without improving the detection rate [20]. In the present study we report on the performance of the Xpert MTB/RIF on archived GA and IS samples collected on two consecutive days from children with probable intrathoracic TB, recruited as part of a double blinded randomized controlled trial to study the role of micronutrient supplementation in children with intra-thoracic tuberculosis.

## Methods

### Study design and subject recruitment

The study was conducted between January 2009 to December 2012 at the All India Institute of Medical Sciences (AIIMS) and Kalawati Saran Children’s Hospital (KSCH), both in New Delhi, India. This work was part of a double blinded randomized controlled trial to study the role of micronutrient supplementation in children with intra-thoracic tuberculosis [19–22]. A total of 403 children (58.2 % girls), aged between 6 months to 14 years [Median age, 120 months; interquartile range (IQR), 7–168 months], with probable intra-thoracic TB were enrolled. The standard clinic-radiological criteria for suspecting intra-thoracic tuberculosis was used in this study as published earlier [19–22]. The diagnosis of intrathoracic tuberculosis was based on recommendations of Indian Academy of Pediatrics. Any child with cough and fever of more than 2 weeks with no improvement during seven to ten days amoxicillin course and recent unexplained weight loss or history of contact with TB patient in past two years. All tuberculosis suspects underwent a chest x-ray (lateral and posterior-anterior). In presence of persistent x-ray abnormalities, with non-resolution of clinical symptoms and no alternative cause for symptoms and chest x-ray findings, a probable diagnosis of intrathoracic tuberculosis was made. All children with probable tuberculosis were subjected to tuberculin skin test (TST), ambulatory gastric aspirates (GA) and induced sputum (IS) examination for AFB smear and culture. Children were started on ATT as per recommendations of the RNTCP [5]. In brief: All children with probable intra-thoracic tuberculosis were subjected to gastric aspiration (GA) and induced sputum (IS) collection for two consecutive days (D1 & D2). Children were asked to report 6 h fasting in the morning to the designated clinic. After explaining the procedure and obtaining consent from parents and verbal assent from the children, an appropriate sized feeding tube (10-12G) was inserted through one nostril till it reached the stomach. The position of tube was checked by insufflation of air into stomach. The contents of stomach were aspirated completely, kept in sterile container. Usual volume collected was around 10 ml. Samples were transported to laboratory for further processing within 1–2 h.

In the same sitting gastric aspiration was followed by induced sputum with a gap of around half hour. For sputum induction, the child was primed with 2 puffs salbutamol inhalation by metered dose inhaler (MDI Asthalin,100 μg/puff, Cipla, India)) and spacer (Zeroastat VT spacer, Cipla, India) followed by nebulization with 3 mL of 3 % saline over next 15–20 min. All samples were given unique code numbers; and sent to the laboratory without a link to clinical and patient details. Samples were processed on the same day for AFB smear, BACTEC-MGIT culture and *in-house* PCR [23]. Clinical and other patient details were recorded in excel sheet by treating pediatrician and the laboratory was not aware of the clinical diagnosis of the subjects.

### Ethical considerations

The study was approved by Institutional ethics committee of All India Institute of Medical Sciences, New Delhi and Kalawati Saran Children Hospital, New Delhi, India, and the trial was registered at clinicaltrials.gov (NCT 00801606). Written informed consent was obtained from parents/guardians of each child.

### Laboratory methods

#### Sample processing

IS and GA (pH non-neutralized) samples were processed as described previously [19, 21, 23, 24]. In brief, the samples were centrifuged at 10,000 x g for 10 min, at 4 °C; supernatant decanted and pellet was re-suspended in an equal volume of 0.5 % NALC–4 % NaOH mixture in 50 ml ridge capped round bottom processing tubes. The suspension was thoroughly (4 x 10 s each interval) vortexed to ensure proper mixing and was incubated at 37 °C for 10 min. After incubation, alkaline pH was neutralized with phosphate buffer [PBS (pH 6.8, total volume of 50 ml)] followed by final centrifugation at 10,000 x g for 10 min, at 4 °C. The supernatant was decanted; the pellet re- suspended in 2 ml of the PBS and 0.5 ml volume aliquoted in 4 sterile tubes. One aliquot was used for smear preparation, one for culture and another for testing in an *in-house* multiplex PCR (m-PCR). One aliquot was stored at −80 °C for further use. From one aliquot, a 500 μl suspension was inoculated in BACTEC MGIT-960 liquid culture system (BACTEC^TM^ MGIT, Becton Dickinson, Sparks, USA), using the standard protocol [25]. Irrespective of a positive or a negative flashed result, all cultures were examined by Ziehl-Neelsen staining. Differentiation of isolates into *Mycobacterium tuberculosis* (MTB) or non-tuberculous mycobacteria (NTM) was undertaken by a species specific *in-house* m-PCR [23]. The IS and GA sample aliquots, stored at −80 °C, were retrospectively examined in the Xpert MTB/RIF assay.

### Xpert MTB/RIF Test

In 2013, the stored samples were subjected to Xpert MTB/RIF testing. There were 520 decontaminated GA and IS paired samples (130x2 GA & 130x2 IS) that were suitable for testing, while others were either not sufficient in quantity or quality (contaminated). The sample reagent buffer containing NaOH and isopropanol was added in the ratio of 3:1 (total 2 ml), and incubated for 15 min at room temperature. The treated sample (2 ml) was then transferred into the Xpert MTB/RIF cartridge containing the wash buffer, lyophilized reagents for DNA extraction and PCR amplification. After proper mixing, the cartridge was loaded in the Xpert MTB/RIF instrument, as per the manufacturer’s instructions (Cepheid, USA). The instrument performs specimen mixing, sonication of the mycobacterial bacilli and internal control (spores), DNA release and mixing with the PCR reagents, automatically. This is followed by a hemi-nested real time-PCR amplification, target detection by five-color fluorescence molecular beacon probes and internal control *in-situ*. Results are generated after 2 h and reported as *M. tuberculosis* - negative or positive with semi-quantified bacillary load as high, medium, intermediate or low and if the pathogen is RIF sensitive or resistant [14]. The patient details were linked to the samples tested only after completion of the Xpert MTB/RIF evaluation.

## Statistical analysis

Sensitivity of Xpert MTB/RIF was determined by considering MGIT culture confirmed samples as true positive. To determine the specificity of Xpert MTB/RIF, smear, MGIT culture and *in-house* m-PCR negative samples were considered as true negative samples. Positive predictive values (PPV), negative predictive value (NPV) and likelihood ratio for positive (LRP) Xpert MTB/RIF were calculated with 95 % CI (confidence intervals). LRP of greater than one indicates that the test result is most likely true positive and less than one means that the test result is likely true negative [26]. Pearson chi square test was performed for comparison of patient wise analysis. To rule out the proportion of agreement by chance, Cohen’s *kappa* test was used. A p-value of <0.05.was considered statistically significant [STATA 11.0 (college station, Texas, USA) software was used for all statistical analysis].

## Results

**Fig. 1:**
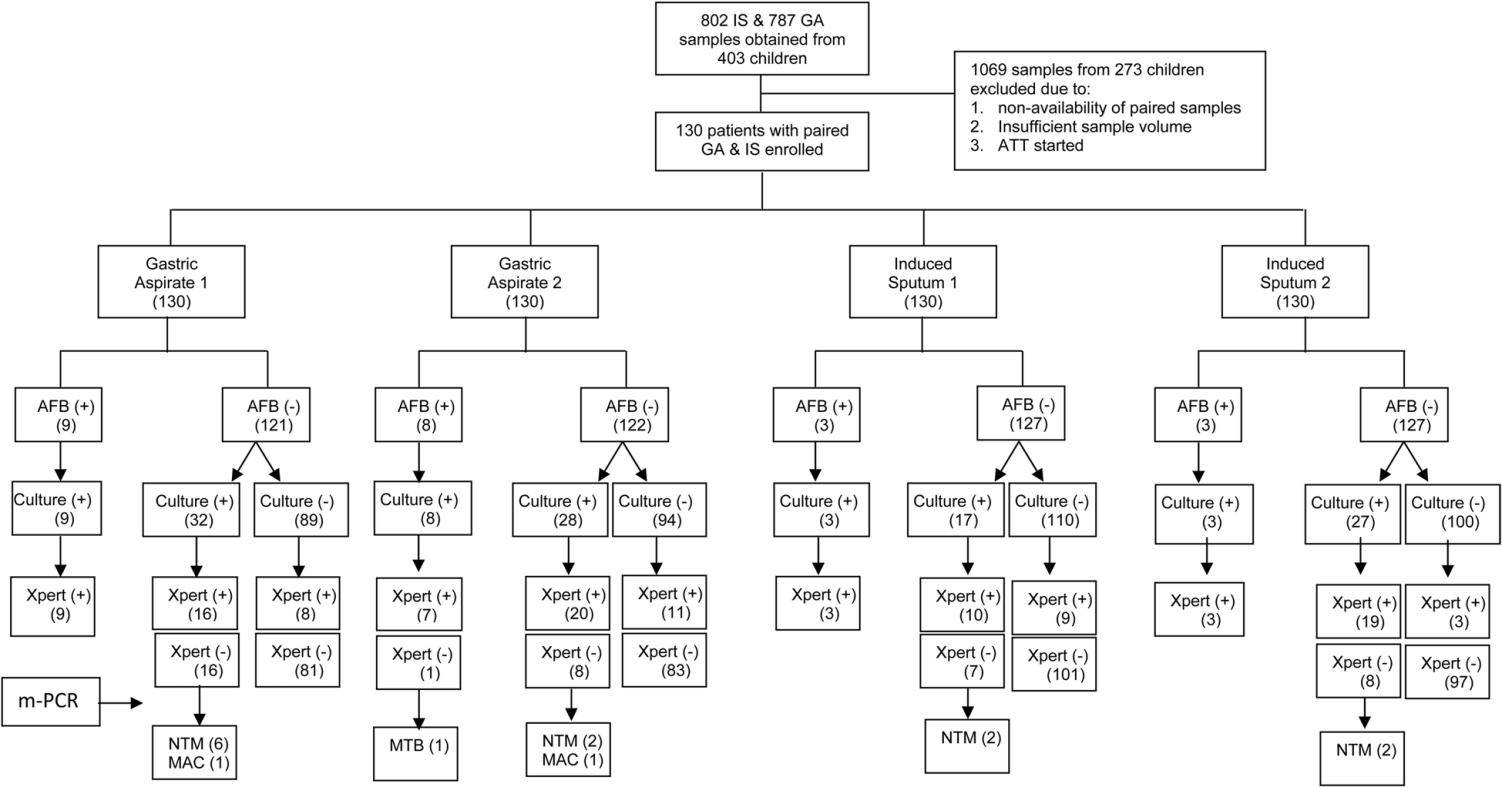
Flow chart depicting the inclusion of samples with flow of sample processing, methods used and results as per STROBE guidelines

In this study, conducted between January 2009 to December 2012. A total of 403 children (58.2 % girls), aged between 6 months to 14 years [Median age, 120 months; interquartile range (IQR), 7–168 months], with probable intra-thoracic TB were enrolled. Only those children were included from whom both GA and IS samples collected on two consecutive days were available. Therefore, paired IS and GA samples obtained on two consecutive days from 130 children were analysed by Xpert MTB/RIF assay. The decontaminated samples were stored at −80 °C for 21.4 to 51.9 months (median storage period 32.5 months; IQR 27–35.1) before these were subjected to Xpert MTB/RIF analysis. The laboratory workflow and summary of results are shown in Fig. 1.

### Diagnostic outcome of IS and GA samples collected on two consecutive days

**Fig. 2:**
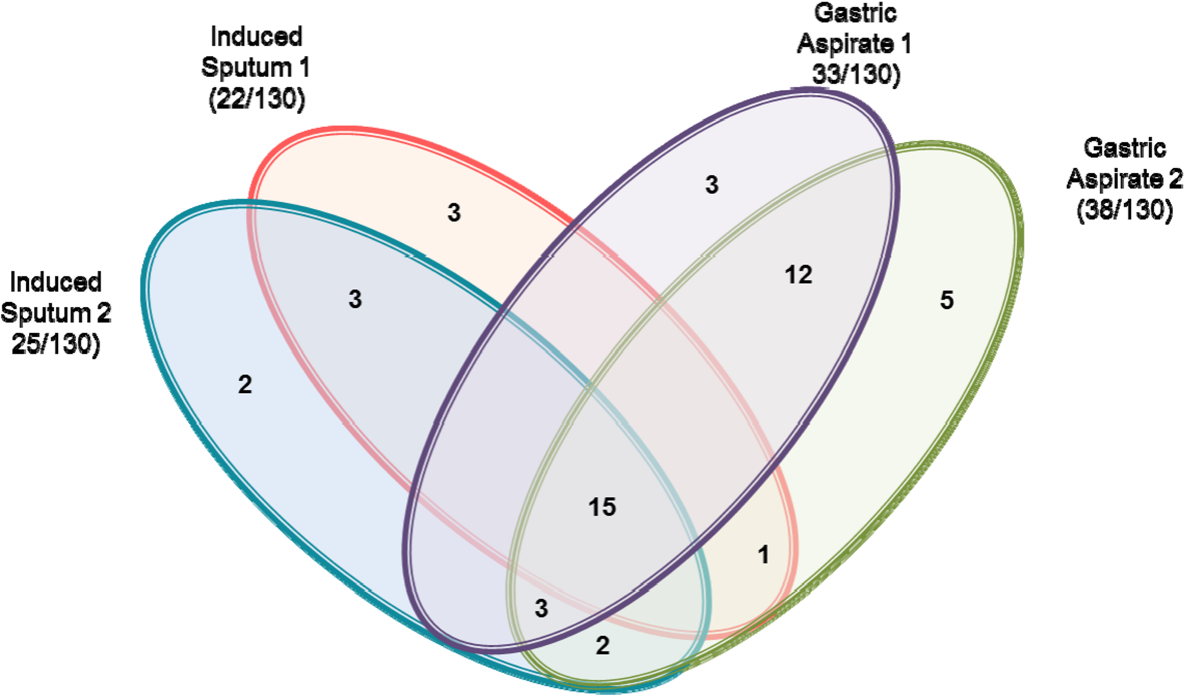
Individual and overlap analysis of Xpert MTB/RIF positivity in gastric aspirate and induced sputum samples collected on 2 consecutive days

Only 23 out of 520 (4.4 %) samples from 13 (10 %) children were AFB smear positive (Fig. 1). Of these 23 samples, 9 were D1 and 8 were D2 GA samples. Second day (D2) GA samples detected 3 additional AFB smear positive cases while it missed 4 cases which were positive on D1, hence there was no net incremental value from a D2 GA sample. Only 3 IS samples were positive on either D1 or D2. The D2-IS sample added one case but also missed one case. Sixty one out of 130 (46.9 %) children [127 of 520 (24.4 %) samples] were diagnosed to have culture confirmed tuberculosis either by smear or culture (Table 1). Out of 130 D1-GA samples 41 (31.5 %) while 36 (27.7 %) of D2-GA samples were culture positive. Twenty three (17.7 %) children had a culture positive GA on both days (Table 2). The yield from induced sputum samples was poor. Of 130 IS samples collected on D1, only 20 samples (15.4 %) were positive while on D2, 30 (23.1 %) samples were culture positive (Table 3). Sixteen (12.3 %) children had a culture positive IS on both days. The MGIT culture yields from GA and IS samples collected on two consecutive days is shown in (Tables 2 & 3, respectively). There was no additional value of a second day GA sample for smear microscopy (9 vs 8), culture (41 vs 36), or Xpert MTB/RIF assay (33 *vs* 34). However, IS samples collected on two consecutive days provided a significant additional value in culture (20 *vs* 30) but an insignificant additional increment in the Xpert MTB/RIF assay (22 *vs* 25). A combination of one GA and one IS sample gave best diagnostic utility, irrespective of D1 or D2. Almost, 50 % samples (either GA or IS) were Xpert MTB/RIF positive on both days (Fig. 2).

**Table 1:** Performance of Xpert MTB/RIF assay in bacteriologically positive and bacteriologically negative samples [combination of gastric aspirates (*n* = 260) and induced sputa (*n* = 260)] collected from children with probable intrathoracic tuberculosis

Bacteriological criteria (N = 520)	Sub-criteria	No. (%)	Xpert MTB/RIF Results	
			Positive (%)	Negative (%)
Bacteriologically positive (*n* = 127; 24.4 %)	Smear (+), culture (+)	23 (4.4)^*, **^	22 (95.7)	1 (4.3)
	Smear (−), culture (+)	104 (20)^*^	65 (62.5)	39 (37.5)
	Smear (+), culture (−)	0	0	0
	Subtotal	127	87 (68.5)	40 (31.5)
Bacteriologically negative (*n* = 393; 75.5 %)	Smear (−), culture (−)	393	31 (7.9)	362 (92.1)
	Total	520	118 (22.7)^**^	402 (77.3)

The table shows high efficacy of Xpert MTB/RIF in culture and smear positive samples but moderate detection rate in smear negative culture positive samples

^*,**^ *P* < 0.001, (+)- positive, (−)- Negative, number in parenthesis indicate percentage

**Table 2:** Performance of MGIT 960 culture on gastric aspirate samples collected on two consecutive days from 130 children with probable intrathoracic tuberculosis

MGIT960 Culture Results		Day 1		
		Positive	Negative	Total
Day 2	Positive	23 (17.7 %)	13 (10 %)	36 (27.7 %)
	Negative	18 (13.8 %)	76 (58.5 %)	94 (72.3 %)
	Total	41 (31.5 %)	89 (68.5 %)	130

**Table 3:** Performance of MGIT 960 culture on induced sputum samples collected on two consecutive days from 130 children with probable intrathoracic tuberculosis

MGIT 960 Culture Results		Day 1		
		Positive	Negative	Total
Day 2	Positive	16 (12.3 %)	14 (10.8 %)	30 (23.1 %)
	Negative	4 (3.1 %)	96 (73.8 %)	100 (76.9 %)
	Total	20 (15.4 %)	110 (84.6 %)	130

### Comparison of IS and GA

Overall, in samples (IS + GA) which were positive either in smear and/or culture (127/520), the sensitivity of Xpert MTB/RIF assay was slightly lower (68.5 %, 87/127) as compared to bacteriological examination (Table 1) but it also detected 7.9 % (31/393) additional cases over the culture giving an overall sensitivity of 22.7 % [118/520] (*p* = NS) but significantly higher (*p* = 0.001) sensitivity than smear microscopy (Table 4). The performance of Xpert MTB/RIF was 95.6 % (22/23) on smear and culture positive archived samples, as shown in (Table 1) and flow chart (Fig. 1). All 14 samples that yielded NTM species were smear negative but culture and m-PCR positive. Hence on re-evaluation, the sensitivity of Xpert MTB/RIF on smear negative but culture positive (MTB only) samples was 72.2 % (65/90). But if all culture positive samples were taken together without considering the mycobacterial species, the sensitivity fell down to 62.5 % (65/104) due to 14 NTM isolates. The sensitivity of Xpert MTB/RIF did not differ much between the GA and IS samples if all bacteriologically (smear and /or culture) positive GA (92.2 %, 71/77) and IS (94 %, 47/50) samples were compared. No NTM positive sample was falsely identified by Xpert MTB/RIF but it missed one smear, culture and m-PCR positive sample, the isolate was identified as MTB by m-PCR and . All Xpert MTB/RIF positive samples were also m-PCR positive. Hence our in-house m-PCR and Xpert MTB/RIF showed 100 % concordance in NTM/MTB differentiation (Fig. 1).

**Table 4:** Sensitivity, specificity and predictive values of Xpert MTB/RIF assay in reference to AFB smear and Bactec-MGIT960 culture in induced sputum (*n* = 260) vs. gastric aspirate (*n* = 260) samples

	Sensitivity	Specificity	PPV	NPV	LRF of positive test	Diagnostic accuracy	Sensitivity (smear positive)	Sensitivity (smear negative)
Xpert MTB/RIF assay vs Culture								
Induced Sputum	35/50 (70.0 %; 56.2–80.9)^*^	198/210 (94.3 %; 90.3–96.7)^**^	74.5 % (60.5–84.6)	92.7 % (88.7–95.7)	12.2 % (10.2–14.8)	89.6 % (85.3–92.8)	6/6 (100 %)	29/44 (65.9 %)
Gastric Aspirate	52/77 (67.5 %; 56.5–76.9)^*^	164/183 (89.6 %; 84.3–93.2)^**^	73.2 % (61.9–82.1)	87.8 % (81.2–90.9)	6.5 % (5.8–7.3)	83.1 % (78.0–87.1)	16/17 (94.1 %)	36/60 (60.0 %)
AFB vs Culture								
Induced Sputum	6/50 (12.0 %; 5.6–23.8)^***^	210/210 (100 %; 98.2–100)	100 % (60.8–100)	82.7 % (77.5–86.8)	-	83.1 % (78.1–87.1)	NA	NA
Gastric Aspirate	17/77 (22.1 %; 14.3–32.5)	183/183 (100 %; 97.9–100)	100 % (81.6–100)	75.3 % (71.4–81.6)	-	76.9 % (71.4–81.6)	NA	NA

Data n/N (%; 95 % CI) or % (95 % CI), Positive predictive value (PPV), negative predictive value (NPV), Likelihood ratio (LRF), ^*^ *p* = 0.77, ^**^ *p* = 0.087, ^***^ *p* = 0.792

### Comparison of Xpert MTB/RIF, Culture and AFB smear microscopy

**Fig. 3:**
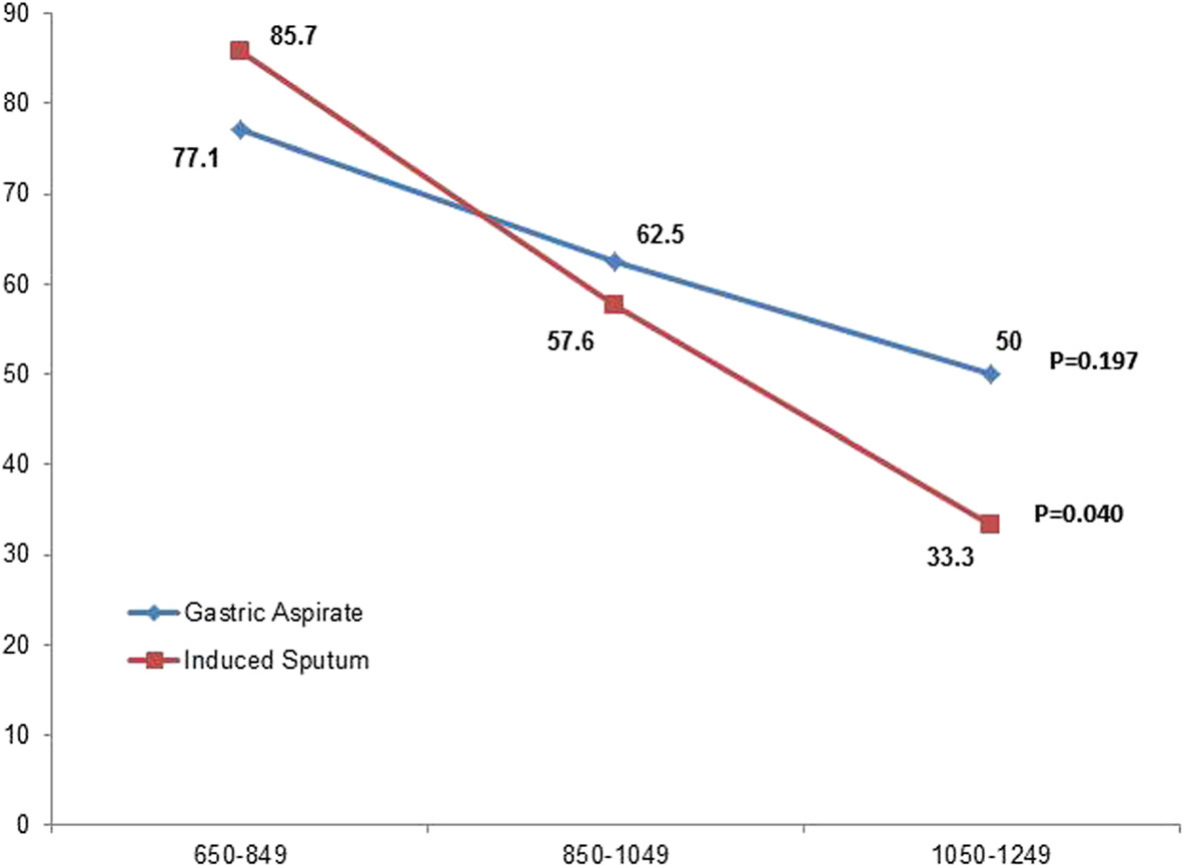
Declining positivity rates in the Xpert MTB/RIF assay in relation to duration of storage of gastric aspirate and induced sputum samples

Xpert MTB/RIF assay detected an additional 31 of 393 (7.9 %) samples which were both AFB smear and MGIT culture negative but missed 39 of 104 (37.5 %) culture positive samples (Table 4). In children with probable intrathoracic tuberculosis, gastric aspirate identified more cases than induced sputum using smear microscopy, culture and Xpert MTB/RIF assay (6.5, 29.6, and 27.3 % *vs* 2.3, 19.2 and 18.0 %, respectively). Yield of Xpert MTB/RIF assay decreased with increasing duration of storage of GA as well as IS samples. In samples stored for 650–849 days, the detection rate of Xpert MTB/RIF assay on IS samples was 85.7 % which dropped to 33.3 % in samples stored for more than 1050 days. Similarly, these rates were 77.1 and 50 %, respectively in GA samples (Fig. 3). After long storage difference in the yield of IS and GA was highly significant (*P* = 0.002).

## Discussion

There has been a flow of publications on Xpert MTB/RIF also known as GeneXpert or cartridge based nucleic acid amplification testing (CBNAAT) in the last 3 years. This is the first study from India on the use of the Xpert MTB/RIF assay in children with intrathoracic TB and also the first study to report on the utility of this assay on archived GA and IS samples from children in TB endemic country. The study shows that even in GA and IS samples stored for up to 4 years, the Xpert MTB/RIF assay remained useful as a diagnostic tool for childhood tuberculosis. In GA and IS samples which were smear and culture positive, the sensitivity of Xpert MTB/RIF assay was 95.6 %, which is similar to that reported in fresh clinical samples. As expected the sensitivity of Xpert MTB/RIF was superior to that of smear microscopy (*p* = 0.0001). However, the sensitivity of Xpert MTB/RIF assay on culture positive but smear negative samples was lower (62.5 %). Performance of Xpert MTB/RIF assay has previously been evaluated mostly on sputum samples collected from adult TB patients [27–30], showing a high sensitivity in smear and culture positive samples (98–100 %) but a much lower sensitivity in smear negative sputum samples (43–70 %). Our findings were also similar to these findings, but on archived samples. A lower sensitivity of the Xpert MTB/RIF assay has also been reported in HIV positive adult patients [29]. There are few studies that have been conducted in children and a very few where performance of Xpert MTB/RIF assay has been evaluated on samples other than sputum samples [4–10, 30, 31]. Nicol et al. [8] included 452 children for analysis with at least one IS sample from each child. They also showed a poor sensitivity of microscopy (6 %), culture (16 %) and Xpert MTB/RIF assay (13 %) in these samples. In the present study, though detection rates of these three methods were higher but the pattern was similar. Since we examined both IS and GA samples on two consecutive days, on comparison the yield from IS samples was much lower than gastric aspirate samples. Though there is no data published so far in which both GA and IS samples have been used in the same study to evaluate the sensitivity and specificity of Xpert MTB/RIF assay in clinically probable cases of intrathoracic tuberculosis, yet our results were in total concordance with findings of Bates et al. [10], Rachow et al. [16] and Sekadde et al. [17]. Our study shows that smear and culture yields in GA samples are superior to those of IS samples, however, the sensitivity of Xpert MTB/RIF assay was not significantly different in GA and IS samples, in samples stored at −80 °C for the same duration (77.1 and 85.7 %, respectively; *P* = 049). Furthermore, we collected both these samples on two consecutive days, and this protocol slightly provided better yield in all diagnostic techniques i.e. smear, culture and Xpert MTB/RIF assay. Because of this double pronged approach, we could achieve a high diagnostic {61 of 130; 46.9 % children (per patient analysis)} yield.

Most publications on Xpert MTB/RIF or CBNAAT have been from Sub-Saharan Africa [6, 8–10, 13, 16, 17]. India is a TB high burden country and the central TB division of India, which has been bestowed the responsibility of implementing the revised national TB control programme has recently installed more than 80 CBNAAT machines.. But country specific data and guidelines on using CBNAAT machines for diagnosing childhood tuberculosis are lacking. Our is also the first study wherein we have used gastric aspirate and induced sputum sample stored for upto 4.3 years (median storage period of 32.5 months). In smear and culture proven samples it showed sensitivity of 95.7 % which was within the range reported by several other workers but only on fresh samples [10, 17]. In fresh samples a variable value addition of the Xpert MTB/ RIF over the MGIT culture has been reported, but in our study this value addition was minimal (7.9 %), probably due to some loss of mycobacterial viability on long-term storage of samples [32]. The additional cases detected over the gold standard culture test, were very unlikely to be false positive. Mycobacterial counts deplete on storage of samples even after few days (30) but contradictory observations have also been made by other workers [33, 34]. Paramasivan et al. [33] reported that there was no effect on the sensitivity rates of smear and culture after storing the sputum samples upto 10 days. Tessema et al. [34] have reported that storage of sputum samples upto 80 days at room temperature and in frozen samples upto 180 days had no negative effect on culture yield. Banda et al. [35] observed that sputum samples remained smear positive upto 8 weeks, and culture positivity was 54–67 % after 4 weeks of storage under frozen conditions but the positivity rate went down to 37–39 % if the samples were stored at room temperature. There are no previous reports on the effect of long term storage on the sensitivity of Xpert MTB/RIF on GA and IS samples. Smear positive samples carry high bacillary (>10000 bacilli /ml) counts, which presumably means that samples having a count of more than 104 bacilli/ml will have sufficient counts detectable by Xpert MTB/RIF even after several years. TB control programmes can therefore, retrospectively trace the real-time incidence and rise in MDR-TB rates in specific areas or countries, using the exact dates of archiving the samples and correlating the epidemiological pattern and emergence of MDR-TB in those countries. The results from this study can contribute to providing such a data for India. Furthermore, this study is the first to evaluate the Xpert MTB/RIF on a large number of representative samples which include a paired GA and IS sample collected on two consecutive days on children with intrathoracic TB.

Xpert MTB/RIF showed high specificity for NTM positive samples as did our *in-house* PCR. But in comparison with the *in-house* m-PCR, Xpert MTB/RIF missed one *M. tuberculosis* isolate (flow chart). We are analyzing this isolate, to determine if the isolate has a unique mutation in *rpoB* gene which inhibited amplification of this gene [36]. We conclude that the Xpert MTB/RIF assay is reliable test for rapid and correctly diagnosing pulmonary tuberculosis in children using GA and IS samples, preferably in multiple samples. Furthermore, the assay can also be used in retrospective testing of samples stored for at least 4.5 years with acceptable diagnostic sensitivity and detection of RIF resistance.

## Conclusions

Combination of 2 gastric aspirates and 2 induced sputum samples collected on two consecutive days, and three diagnostic methods i.e. smear examination, liquid culture and Xpert MTB/RIF, yielded a very high TB detection rate. Overall and per patient detection rate of BACTEC-MGIT culture was 24.4 and 46.9 %, respectively. Though there was no significant difference in the diagnostic yield of samples collected on day 1 and day 2, but a combination of GA and IS yielded best results. Despite long term storage, the overall sensitivity of Xpert MTB/RIF on smear and -culture positive samples were 95.6 %. In smear negative and culture positive samples the sensitivity of Xpert MTB/RIF was 62.5 %. But in bacteriologically confirmed samples, as expected, the detection rate was higher (68.5 %). The TB detection rate of smear microscopy, culture and Xpert MTB/RIF assay was also higher in GA samples than in IS samples. To our knowledge, this is the first report which shows that smear and culture positive samples stored at subfreezing conditions for upto 3.5 years can be used in Xpert MTB/RIF assay without a significant effect on the diagnostic sensitivity and specificity of the test.
